# Diurnal Response of Photosystem I to Fluctuating Light Is Affected by Stomatal Conductance

**DOI:** 10.3390/cells10113128

**Published:** 2021-11-11

**Authors:** Ting-Yu Li, Qi Shi, Hu Sun, Ming Yue, Shi-Bao Zhang, Wei Huang

**Affiliations:** 1 School of Life Sciences, Northwest University, Xi’an 710069, China; litingyu@mail.kib.ac.cn (T.-Y.L.); yueming@nwu.edu.cn (M.Y.); 2 Kunming Institute of Botany, Chinese Academy of Sciences, Kunming 650201, China; shiqi@mail.kib.ac.cn (Q.S.); sunhu19@mails.ucas.ac.cn (H.S.); 3 University of Chinese Academy of Sciences, Beijing 100049, China

**Keywords:** electron transport, fluctuating light, photosynthesis, photosystem I, stomatal conductance

## Abstract

Upon a sudden transition from low to high light, electrons transported from photosystem II (PSII) to PSI should be rapidly consumed by downstream sinks to avoid the over-reduction of PSI. However, the over-reduction of PSI under fluctuating light might be accelerated if primary metabolism is restricted by low stomatal conductance. To test this hypothesis, we measured the effect of diurnal changes in stomatal conductance on photosynthetic regulation under fluctuating light in tomato (*Solanum lycopersicum*) and common mulberry (*Morus alba*). Under conditions of high stomatal conductance, we observed PSI over-reduction within the first 10 s after transition from low to high light. Lower stomatal conductance limited the activity of the Calvin–Benson–Bassham cycle and aggravated PSI over-reduction within 10 s after the light transition. We also observed PSI over-reduction after transition from low to high light for 30 s at the low stomatal conductance typical of the late afternoon, indicating that low stomatal conductance extends the period of PSI over-reduction under fluctuating light. Therefore, diurnal changes in stomatal conductance significantly affect the PSI redox state under fluctuating light. Moreover, our analysis revealed an unexpected inhibition of cyclic electron flow by the severe over-reduction of PSI seen at low stomatal conductance. In conclusion, stomatal conductance can have a large effect on thylakoid reactions under fluctuating light.

## 1. Introduction

Fluctuating light (FL) is the typical light condition under natural field conditions [1]. A sudden drop in light intensity will cause a decrease in CO_2_ assimilation rate without photoinhibition [2,3]. By contrast, when the light intensity abruptly increases, the resulting rapid rise in photosystem II (PSII) electron flow is accompanied by relatively slower kinetics of CO_2_ assimilation [4]. As a result, ATP and NADPH produced by linear electron flow cannot be immediately consumed by the primary metabolism. Such imbalance between light and dark reactions then leads to the accumulation of electrons in PSI, which manifests as PSI over-reduction [5,6,7]. Under such conditions, the transfer of electrons from PSI electron carriers to oxygen (O_2_) increases, producing reactive oxygen species (ROS) [8]. Because antioxidant systems cannot immediately scavenge the ROS generated within PSI [9], the FL-induced over-reduction of PSI causes significant PSI photoinhibition [6,10,11,12]. Owing to the important role of PSI in the operation of photosynthetic electron transport, PSI photoinhibition significantly suppresses CO_2_ assimilation, photoprotection, and plant growth [13,14,15,16].

To protect PSI under FL, plants have evolved alternative electron flow pathways to partially alleviate PSI over-reduction. In general, cyclic electron flow (CEF) around PSI is thought to be the main player regulating the PSI redox state under FL [8]. During CEF around PSI, electrons from ferredoxin are transported to the plastoquinone pool and are mediated by the PROTON GRADIENT REGULATION5 (PGR5)/PGR5-LIKE1 (PGRL1) and NAD(P)H DEHYDROGENASE (NDH) pathways [17,18], which are coupled to proton translocation from the stroma to the thylakoid lumen [19,20,21]. Therefore, CEF generates a proton gradient (ΔpH) across the thylakoid membrane without NADPH production. When transitioning from low to high light, CEF is rapidly activated to help with ΔpH formation [22,23,24], which not only downregulates the oxidation of plastoquinone but also enhances the ATP/NADPH production ratio [25], both of which can prevent uncontrolled PSI over-reduction and thus protect PSI under FL [6]. However, CEF cannot avoid a transient PSI over-reduction under FL in many angiosperms [5,6,26], suggesting that the PSI redox state under FL is also largely affected by the electrons downstream of PSI [27,28,29]. In angiosperms, electrons in PSI can be transported to NADP^+^ and O_2_ [30]. The former is dependent on the operation of primary metabolism including CO_2_ fixation and photorespiration [31], whereas the latter is attributed to the water–water cycle [32]. The activity of the water–water cycle is negligible in most angiosperms [33,34,35], although it can significantly regulate PSI redox state under FL in some plants, such as those in the *Camellia* genus [36], *Bryophyllum pinnatum* [37], and the orchid *Dendrobium officinale* [38]. Therefore, the outflow of electrons in PSI under FL largely relies on the operation of primary metabolism. However, the effect of CO_2_ assimilation on the regulation of PSI redox state under FL is not well known.

Many studies have examined photosynthetic regulation under FL in the model plants Arabidopsis (*Arabidopsis thaliana*) and rice (*Oryza sativa*) grown under constant light [4,6,8,29,39,40]. However, the diurnal photosynthetic regulation under FL in wild plants grown under full sunlight has rarely been investigated. Under natural field conditions, diurnal changes in photosynthetic CO_2_ assimilation rate are largely affected by stomatal conductance [41,42,43]. Stomatal conductance determines the extent of CO_2_ diffusion from the air to the intercellular space. A decrease in stomatal conductance lowers the total CO_2_ diffusion conductance, thus restricting CO_2_ fixation owing to the low CO_2_ concentration in chloroplasts [44]. Under such conditions, the consumption of NADPH is blocked, leading to a decrease in the NADP^+^/NADPH ratio. Because the linear electron flow is largely controlled by the NADP^+^/NADPH ratio [45], electron flow from PSI to NADP^+^ at low stomatal conductance is limited by the lack of NADP^+^ [46]. Therefore, when stomatal conductance decreases, the restriction of CO_2_ fixation will suppress the outflow of electrons from PSI to NADP^+^, aggravating PSI over-reduction under FL. Thus, we speculate that stomatal conductance plays an important role in the regulation of the PSI redox state under FL and that the response of PSI to FL is likely affected by diurnal changes in stomatal conductance.

In this study, we measured diurnal photosynthetic regulation under FL in tomato (*Solanum lycopersicum*) and common mulberry (*Morus alba*). The main aims were to (1) determine whether a decrease in stomatal conductance accelerates PSI over-reduction under FL and (2) assess whether the diurnal response of PSI to FL is controlled by stomatal conductance.

## 2. Materials and Methods

### 2.1. Plant Materials

Tomato (*Solanum lycopersicum* Miller cv. Hupishizi) and common mulberry (*Morus alba*) plants were cultivated in full sunlight. The day/night air temperatures were approximately 30 °C/20 °C, and the maximum sunlight intensity at noon was approximately 2000 μmol photons m^−2^ s^−1^ (measured by a Li-1400 datalogger, Li-Cor Biosciences, Lincoln, NE, USA). Seedlings were grown in plastic pots with humus soil with an initial soil N content of 2.1 mg/g. Plants were fertilized with Peter’s Professional water-soluble fertilizer (N:P:K = 15:4.8:24.1) once every 2 days. To prevent any water stress, the plants were watered every day. We used *F*_v_/*F*_m_ to quantify stress. All leaves used for measurements had *F*_v_/*F*_m_ values higher than 0.80. The youngest fully developed leaves were used for measurements.

### 2.2. Experimental Design

In a preliminary experiment, stomatal conductance at 15:00 and 18:00 was measured in tomato leaves, which revealed that stomata were closed at 18:00 relative to 15:00. Furthermore, the photosynthetic response to FL in tomato leaves was significantly different at 15:00 and 18:00, indicating that the PSI redox state under FL can be affected by stomatal conductance. To confirm this finding, diurnal photosynthetic responses to FL were measured in the wild plant *Morus alba*. After measurement of incident stomatal conductance, leaves were exposed to a high light intensity of 1809 (or 1455) μmol photons m^−2^ s^−1^ for 5 min to activate photosynthetic electron sinks. Afterwards, leaves were exposed to FL alternating between 59 and 1809 (or 1455) μmol photons m^−2^ s^−1^ every 2 min. For leaves of *Morus alba*, the light saturation point is approximately 1455 μmol photons m^−2^ s^−1^. For leaves of tomato, 1809 μmol photons m^−2^ s^−1^ is needed for maximum photosynthesis.

### 2.3. Stomatal Conductance Measurements

The diurnal changes in stomatal conductance were measured with a leaf porometer (SC-1 porometer; Decagon Devices, Inc., Pullman, WA, USA) on intact leaves. The incident stomatal conductance was measured under ambient sunlight.

### 2.4. PSI and PSII Measurements

We used a Dual-PAM 100 measuring system (Heinz Walz, German) to measure PSI and PSII parameters under atmospheric CO_2_ conditions [47]. The 635 nm actinic illumination from an LED array was used as the light source. A 5 min dark incubation was used to measure *P*_m_. The PSI parameters were calculated as follows: Y(I) = (*P*_m_’ − *P*)/*P*_m_; Y(ND) = *P*/*P*_m_; Y(NA) = (*P*_m_ − *P*_m_’)/*P*_m_. Y(I), the quantum yield of PSI photochemistry; Y(ND), the quantum yield of PSI non-photochemical energy dissipation due to donor side limitation; Y(NA), the quantum yield of PSI non-photochemical energy dissipation due to acceptor side limitation. The effective quantum yield of PSII photochemistry was calculated as Y(II) = (*F*_m_’ − *F*_s_)/*F*_m_’. The relative photosynthetic electron transport rate through PSI and PSII was calculated as: ETRI = PPFD × Y(I) × 0.84 × 0.5; ETRII = PPFD × Y(II) × 0.84 × 0.5. ETRI minus ETRII was assumed to be the rate of CEF.

### 2.5. Statistical Analysis

All results are shown as mean values of five individual experiments. Tukey’s multiple comparison test was used to determine the significant differences between different treatments (*α* = 0.05). The software SigmaPlot 10.0 was used for graphing and fitting.

## 3. Results

### 3.1. A Decrease in Stomatal Conductance Induces PSI Over-Reduction under FL in Tomato

**Figure 1:**
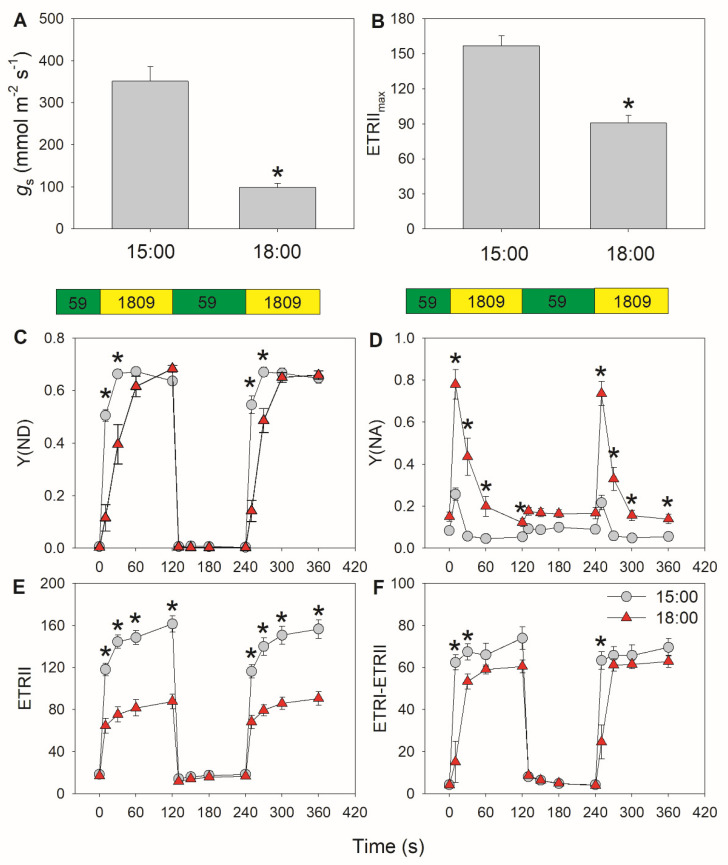
Assessment of diurnal photosynthetic performance in tomato leaves measured at 15:00 and 18:00: (**A**) stomatal conductance (*g*_s_); (**B**) maximum electron transport rate through PSII (ETRII_max_); (**C**–**F**) changes in photosynthetic parameters during fluctuating light alternating between 59 and 1809 μmol photons m^−2^ s^−1^; (**C**) Y(ND), quantum yield of PSI non-photochemical energy dissipation due to the donor side limitation; (**D**) Y(NA), quantum yield of PSI non-photochemical energy dissipation due to the acceptor side limitation; (**E**) ETRI, electron transport rate through PSI; (**F**) estimated CEF performance, calculated as ETRI–ETRII. Asterisk indicates a significant difference between 15:00 and 18:00 samples. Data are shown as mean ± standard error (SE, *n* = 5).

We measured the photosynthetic responses to FL at 15:00 and 18:00 in tomato leaves. The stomatal conductance at 18:00 was much lower than that at 15:00 (Figure 1A), which was accompanied by a decrease in maximum PSII electron flow (ETRII_max_) under FL (Figure 1B). Within 10 s after the transition from 59 to 1809 μmol photons m^−2^ s^−1^, the quantum yield of PSI non-photochemical energy dissipation due to the donor side limitation (Y(ND)) increased from 0 to approximately 0.5 at 15:00, but only reached approximately 0.1 at 18:00 (Figure 1C). This moderate rise in Y(ND) at 18:00 led to severe PSI over-reduction, as indicated by the high value measured for the quantum yield of PSI non-photochemical energy dissipation due to the acceptor side limitation (Y(NA)) (Figure 1D). Y(NA) then decreased to its lowest value starting 30 s after the light transition at 15:00, but remained high at 18:00. These results indicated that the PSI redox state under FL changed during diurnal photosynthesis. When stomata were closed at 18:00, ETRII was not affected under low light but decreased by half under high light compared with values obtained at 15:00 (Figure 1E). To analyze the effect of stomatal closure on CEF performance under FL, we evaluated CEF by subtracting ETRII from ETRI in accordance with established published methods [48,49], although Y(I) can be under/over-estimated depending on the redox state of PC [50,51]. Under low light, CEF activation did not differ between 15:00 and 18:00. However, after transition from low to high light for 10 s, CEF rapidly increased to reach its maximum value at 15:00, but showed a much more modest rise at 18:00 (Figure 1F). CEF required a full 30 s after the transition to increase to its peak value at 18:00. Therefore, lower stomatal conductance substantially influenced the activation speed of CEF under FL.

**Figure 2:**
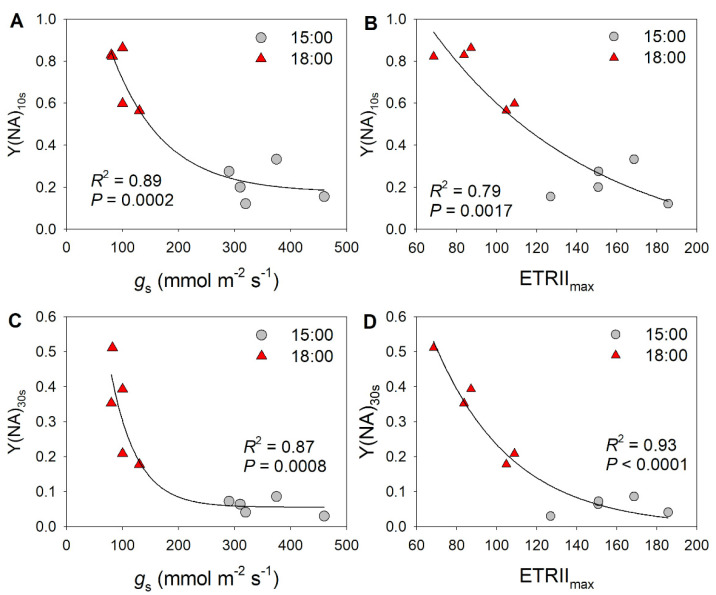
Exploration of correlations between PSI redox state and photosynthetic parameters: relationship between (**A**) stomatal conductance (*g*_s_) and Y(NA) after transition from low to high light for 10 s (Y(NA)_10 s_), between (**B**) maximum electron transport rate through PSII (ETRII_max_) and Y(NA)_10 s_, between (**C**) stomatal conductance (*g*_s_) and Y(NA) after transition from low to high light for 30 s (Y(NA)_30 s_), and between (**D**) ETRII_max_ and Y(NA)_30 s_ in tomato leaves. The data for Y(NA) were taken from the second low/high light cycle, as shown in Figure 1.

**Figure 3:**
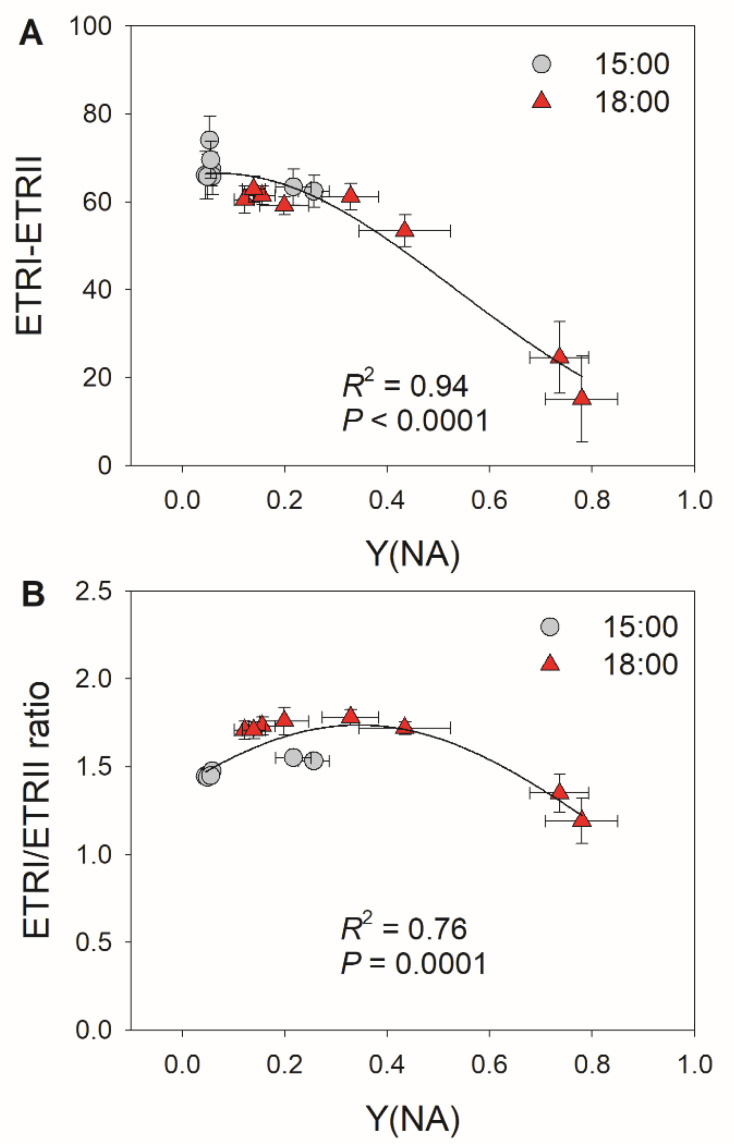
Relationships between (**A**) Y(NA) and ETRI–ETRII and between (**B**) Y(NA) and the ETRI/ERTII ratio during the high light phases under fluctuating light in tomato leaves. Data are shown as mean ± SE (*n* = 5).

Because the decreases in stomatal conductance and ETRII were accompanied by variation in Y(NA) under FL, we examined the relationships between stomatal conductance, ETRII, and Y(NA) after transition from low to high light for 10 and 30 s. As shown in Figure 2, low levels of stomatal conductance and ETRII_max_ were associated with an increase in Y(NA) within the first 30 s after transition from low to high light. With the increases in stomatal conductance and ETRII_max_, PSI over-reduction was gradually relaxed to the minimal value of approximately 0.1. These results suggest that restricting CO_2_ fixation significantly aggravates PSI over-reduction under FL. Furthermore, the severe PSI over-reduction observed after transition from low to high light for 10 s at 18:00 inhibited the activation of CEF (Figure 3). Once PSI over-reduction was relaxed, CEF was fully activated. Therefore, the activation of CEF in high-light phases under FL is likely affected by PSI redox state.

### 3.2. Diurnal Response of PSI to FL in Morus alba Is Controlled by Stomatal Conductance

**Figure 4:**
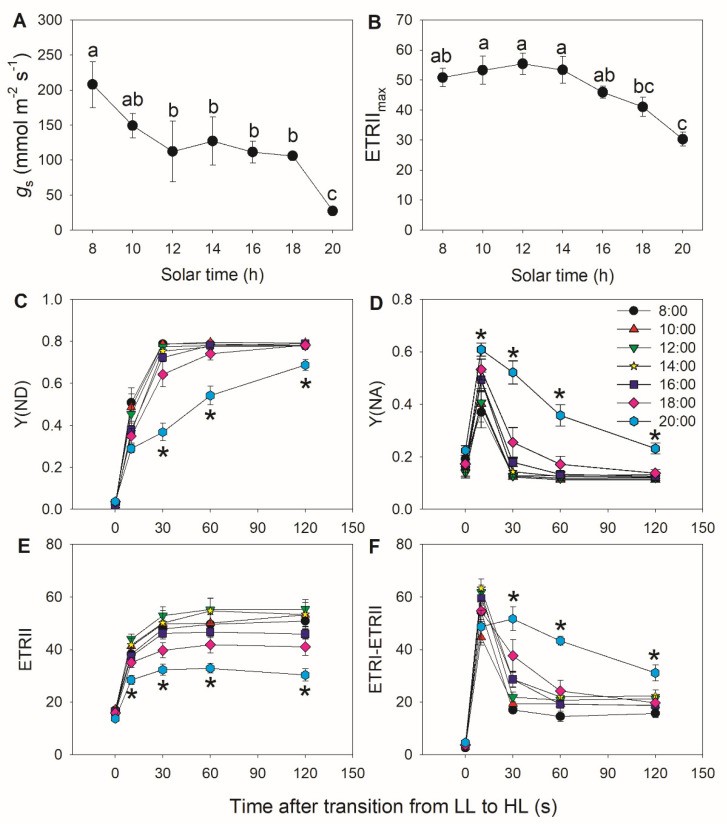
Diurnal variation in photosynthetic performance of *M. alba* leaves: (**A**) stomatal conductance (*g*_s_). (**B**) Maximum electron transport rate through PSII (ETRII_max_). (**C**–**F**) Changes in photosynthetic parameters during fluctuating light alternating between 59 and 1455 μmol photons m^−2^ s^−1^. (**C**) Y(ND), quantum yield of PSI non-photochemical energy dissipation due to donor side limitation. (**D**) Y(NA), quantum yield of PSI non-photochemical energy dissipation due to acceptor side limitation. (**E**) ETRI, electron transport rate through PSI. (**F**) Estimated CEF performance, calculated as ETRI–ETRII. In (**A**) and (**B**), different letters indicate significant differences. In **C**–**F**, asterisk indicates a significant difference between 20:00 and the other times. Data are shown as mean ± SE (*n* = 6).

To confirm the findings obtained in tomato, we examined the diurnal photosynthetic regulation under FL in the wild plant *Morus alba*. Stomatal conductance significantly decreased at 20:00 when compared with other sampling times (Figure 4A) and was associated with the smallest value for ETRII_max_ (Figure 4B). When transitioning from low to high light, Y(ND) did not increase to its maximum value within the first 10 s, but instead required a full 30 s to do so (Figure 4C), leading to the concomitant increase in Y(NA)_10 s_ after the transition (Figure 4D). These results indicate that PSI is over-reduced under FL in *M. alba*. Furthermore, Y(ND) increased much slower at 20:00 than at other time points, resulting in sustained high Y(NA) levels under FL. Therefore, stomatal closure in the late afternoon aggravated PSI over-reduction under FL. The ETRII values after transition to high light decreased in the late afternoon (Figure 4E), suggesting that the CO_2_ assimilation rate is largely restricted by stomatal conductance. After transition to high light, CEF first increased to its peak value after 10 s, which was followed by a gradual decrease to reach its minimal value after 120 s at all time points, except for 20:00 (Figure 4F). The activation of CEF at 20:00 during high light phases was higher than at other time points.

**Figure 5:**
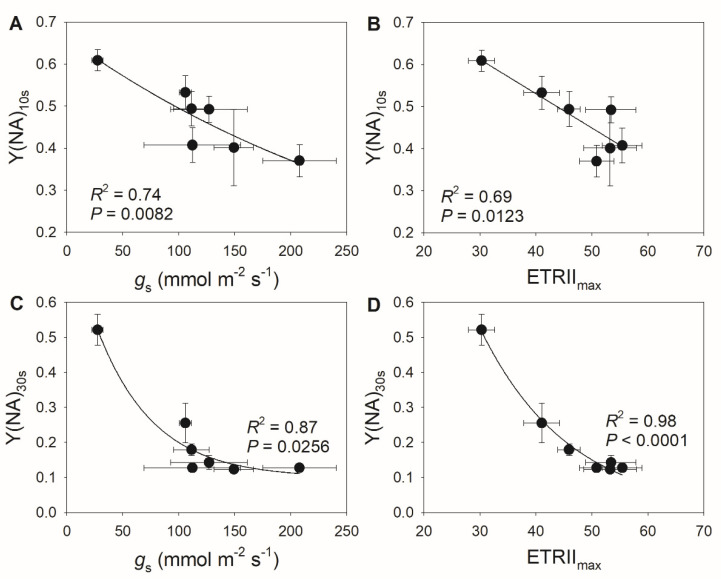
Relationships between (**A**) stomatal conductance (*g*_s_) and Y(NA) after transition from low to high light for 10 s (Y(NA)_10 s_), between (**B**) maximum electron transport rate through PSII (ETRII_max_) and Y(NA)_10 s_, between (**C**) stomatal conductance (*g*_s_) and Y(NA) after transition from low to high light for 30 s (Y(NA)_30 s_), and between (**D**) ETRII_max_ and Y(NA)_30 s_ in *M. alba* leaves. All data are from Figure 4.

**Figure 6:**
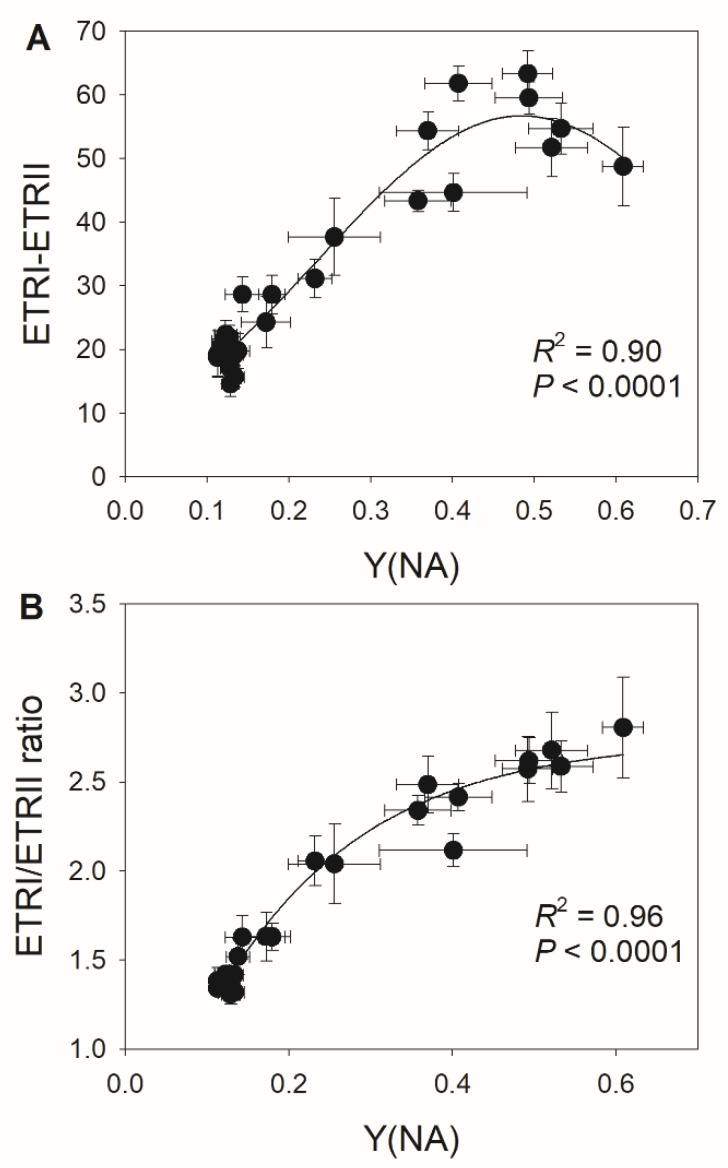
Relationships between (**A**) Y(NA) and ETRI–ETRII and between (**B**) Y(NA) and the ETRI/ERTII ratio during the high light phases under fluctuating light in *M. alba* leaves. Data are shown as mean ± SE (*n* = 6).

We determined that the extent of PSI over-reduction after transition to high light was negatively correlated with stomatal conductance (Figure 5A,B), suggesting that the diurnal response of PSI to FL is largely driven by stomatal conductance. Furthermore, we detected negative relationships between ETRII_max_ and Y(NA) during the high light phase (Figure 5C,D), indicating that the light use efficiency significantly influences the PSI redox state under FL. These results suggest that stomatal conductance has a large effect on the PSI redox state under FL by modulating light use efficiency. During high light phases, CEF activation was strongly and positively correlated with the PSI redox state (Figure 6A), as a higher Y(NA) was accompanied by higher CEF activation. When PSI was over-reduced after the transition to high light, the contribution of CEF to total photosynthetic electron transport rose (Figure 6B), which favored the formation of ΔpH and alleviated PSI over-reduction. Once PSI over-reduction was fully relieved, the CEF activation state decreased, which prevented over-acidification of the thylakoid lumen. Therefore, the contribution of CEF to total photosynthetic electron transport under FL changed diurnally according to the incident PSI redox state in a given condition.

## 4. Discussion

After transitioning from low to high light, tomato and *M. alba* leaves displayed a significant transient over-reduction of PSI (Figure 1D and Figure 4D), indicating that the electrons transported to PSI failed to be immediately consumed by the downstream sinks of PSI. Furthermore, this suggested that the water–water cycle had a negligible contribution in tomato and *M. alba*, as it rapidly consumed the excess reducing power of PSI [11,36,38]. Therefore, the electrons transported from PSII to PSI are mainly channeled toward CO_2_ fixation and photorespiration. During diurnal changes in photosynthetic rates, the decrease in stomatal conductance was accompanied by lower PSII electron flow (Figure 1 and Figure 4), indicating that CO_2_ assimilation was restricted at low stomatal conductance. Under these conditions, the extent of PSI over-reduction within the first 10 s after transition from low to high light was aggravated. Furthermore, when stomatal conductance decreased in the late afternoon, we also observed PSI over-reduction under FL after transition from low to high light for 30 s. These results suggested that the restriction of CO_2_ assimilation by low stomatal conductance significantly induced stronger and prolonged PSI over-reduction under FL. PSI over-reduction increases the transfer of electrons to O_2_, resulting in the generation of ROS in the vicinity of PSI and thus causing PSI photoinhibition [8,9,52,53]. Therefore, PSI susceptibility to photoinhibition under FL can be affected by stomatal conductance.

Stomatal conductance is an important factor determining light use efficiency under FL [54,55,56]. During the diurnal course of photosynthesis, stomatal opening under high-light conditions increases the CO_2_ diffusion efficiency and facilitates CO_2_ assimilation [43,57]. Interestingly, this study determined that stomatal opening could alleviate PSI over-reduction under FL (Figure 2 and Figure 5), which would diminish or prevent FL-induced PSI photoinhibition. When PSI is damaged, photosynthetic electron transport systems including LEF and CEF are suppressed, leading to a lower trans-thylakoid proton gradient and a loss of photoprotection for PSI and PSII [14,16,58]. Photosynthetic CO_2_ assimilation is also restricted by the lack of ATP and NADPH [13,59,60]. Therefore, maintaining PSI activity is a prerequisite for highly efficient CO_2_ assimilation under FL during diurnal photosynthesis [61]. Accordingly, the diurnal stomatal opening under high light is important for protecting PSI under FL and thus guaranteeing optimal photosynthesis over the course of the day.

Recent studies have reported that increased stomatal conductance can enhance both CO_2_ assimilation rate and biomass production under FL in rice and Arabidopsis [43,57], which holds great potential for crop improvement and molecular breeding. A possible explanation for these observations is that the increased conductance lowers the stomatal limitation of photosynthesis by accelerating the diffusion of CO_2_ into leaves, to ensure efficient photosynthetic CO_2_ fixation. In rice and Arabidopsis, PSI is susceptible to photoinhibition under FL, which leads to significant decreases in CO_2_ assimilation rates and biomass when grown under FL [4,6]. Therefore, preventing FL-induced PSI photoinhibition can facilitate sustained high photosynthetic efficiency under FL. In this study, higher stomatal conductance significantly lowered PSI over-reduction under FL (Figure 2 and Figure 5). Therefore, the artificial enhancement of stomatal opening by FL in rice and Arabidopsis will favor PSI photoprotection and thus guarantee photosynthetic reactions, providing a basis for the increase in plant biomass.

Upon transitioning from low to high light, the dynamic flexibility of CEF activity regulates the trade-off between photoprotection and photosynthetic efficiency [30,62]. Within the first seconds after changing from low to high light, CEF was highly activated to help generate the ΔpH necessary for ATP production, which strengthens photosynthetic control of cytochrome *b*_6_*f* and enhances electron transport downstream of PSI [6,10,22]. As a result, this transient CEF stimulation prevents uncontrolled PSI over-reduction under FL [6,8]. When PSI over-reduction is clearly absent, CEF activity decreases to prevent over-acidification of the thylakoid lumen [22,24,63], thus optimizing photosynthetic efficiency [64]. Consistently, we observed that moderate PSI over-reduction under FL induced the greatest CEF activation in both plant species (Figure 3 and Figure 6). Furthermore, we noticed an unexpected inhibition of CEF by severe PSI over-reduction under low stomatal conductance (Figure 3 and Figure 6). When Y(NA) was greater than 0.6, the small number of photo-oxidizable P700 donors in PSI was not sufficient to maintain the normal operation of the CEF. Such an inhibition of the CEF limited ΔpH formation, leading to prolonged PSI over-reduction under FL at low stomatal conductance (Figure 1D and Figure 4D). Therefore, stomatal conductance can have a large effect on thylakoid reactions under FL.

## 5. Conclusions

In this study, we examined the regulation of photosynthesis over the diurnal cycle under FL in tomato and *Morus alba*. The FL-induced over-reduction of PSI was modest at noon but was severe in the late afternoon. Furthermore, the diurnal response of PSI to FL was largely affected by photosynthetic efficiency, which itself was mainly influenced by stomatal conductance. A relatively high stomatal conductance at noon not only ensures efficient photosynthetic CO_2_ assimilation, but also favors PSI photoprotection under FL. Our results provide new insight into the physiological function of stomatal conductance in sustaining photosynthesis over the course of the day.

## Data Availability

The data presented in this study are available on request from the corresponding author.
